# Conservation of Three-Dimensional Helix-Loop-Helix Structure through the Vertebrate Lineage Reopens the Cold Case of Gonadotropin-Releasing Hormone-Associated Peptide

**DOI:** 10.3389/fendo.2017.00207

**Published:** 2017-08-22

**Authors:** Daniela I. Pérez Sirkin, Anne-Gaëlle Lafont, Nédia Kamech, Gustavo M. Somoza, Paula G. Vissio, Sylvie Dufour

**Affiliations:** ^1^Laboratorio de Neuroendocrinología del Crecimiento y la Reproducción, Departamento de Biodiversidad y Biología Experimental, Facultad de Ciencias Exactas y Naturales, Universidad de Buenos Aires, Buenos Aires, Argentina; ^2^CONICET-Universidad de Buenos Aires, Instituto de Biodiversidad y Biología Experimental y Aplicada (IBBEA), Buenos Aires, Argentina; ^3^Muséum National d’Histoire Naturelle, Sorbonne Universités, UMR BOREA, Biologie des Organismes et Ecosystèmes Aquatiques, CNRS, IRD, UPMC, UNICAEN, UA, Paris, France; ^4^Instituto de Investigaciones Biotecnológicas-Instituto Tecnológico de Chascomús (CONICET-UNSAM), Chascomús, Argentina

**Keywords:** GnRH-associated peptide, protein 3D structure, helix-loop-helix, phylogeny, evolution, vertebrates, teleosts

## Abstract

GnRH-associated peptide (GAP) is the C-terminal portion of the gonadotropin-releasing hormone (GnRH) preprohormone. Although it was reported in mammals that GAP may act as a prolactin-inhibiting factor and can be co-secreted with GnRH into the hypophyseal portal blood, GAP has been practically out of the research circuit for about 20 years. Comparative studies highlighted the low conservation of GAP primary amino acid sequences among vertebrates, contributing to consider that this peptide only participates in the folding or carrying process of GnRH. Considering that the three-dimensional (3D) structure of a protein may define its function, the aim of this study was to evaluate if GAP sequences and 3D structures are conserved in the vertebrate lineage. GAP sequences from various vertebrates were retrieved from databases. Analysis of primary amino acid sequence identity and similarity, molecular phylogeny, and prediction of 3D structures were performed. Amino acid sequence comparison and phylogeny analyses confirmed the large variation of GAP sequences throughout vertebrate radiation. In contrast, prediction of the 3D structure revealed a striking conservation of the 3D structure of GAP1 (GAP associated with the hypophysiotropic type 1 GnRH), despite low amino acid sequence conservation. This GAP1 peptide presented a typical helix-loop-helix (HLH) structure in all the vertebrate species analyzed. This HLH structure could also be predicted for GAP2 in some but not all vertebrate species and in none of the GAP3 analyzed. These results allowed us to infer that selective pressures have maintained GAP1 HLH structure throughout the vertebrate lineage. The conservation of the HLH motif, known to confer biological activity to various proteins, suggests that GAP1 peptides may exert some hypophysiotropic biological functions across vertebrate radiation.

## Introduction

Gonadotropin-releasing hormone (GnRH) is a neuropeptide discovered in the 1970s in mammals for its key role in the control of pituitary gonadotropins and reproduction (1, 2). In addition to its hypophysiotropic function on gonadotrophs, including gonadotropin synthesis and release, GnRH has been described as a central and peripheral neuromediator [for review Ref. (3)]. To date, a number of variants of GnRH decapeptide have been identified in vertebrates, and many species express two or three GnRH variants, encoded by distinct genes [for review Ref. (4, 5)]. In vertebrates, GnRH variants are currently classified into three different types, according to their amino acid sequence, localization, embryological origin, and genomic synteny: GnRH1, GnRH2, and GnRH3 [for review: Ref. (6–9)].

The GnRH type ensuring the classical hypophysiotropic function in mammals, as well as in most other gnathostomes, is GnRH1. GnRH1-expressing neurons originate from the olfactory placodes during embryogenesis (10). GnRH1 is the most variable GnRH type according to the decapeptide sequence among vertebrates. GnRH2, also known as “mid brain variant,” was originally discovered in chicken (11) and then described in all vertebrate groups (12). It is the most conserved GnRH type, with an identical GnRH2 decapeptide amino acid sequence in all groups of gnathostomes. Nevertheless, it is not present or it is non-functional in some mammalian species such as mouse, rat, cow, and sheep (10, 13, 14). Regarding to GnRH3, this type, discovered in salmon (15), has been first considered to be present only in teleost fish; however, its origin in early vertebrates and the presence of GnRH variants conforming this clade in some non-teleost species, as in a chondrichtyan (dogfish), a basal sarcopterygian (coelacanth), and a cyclostome (lamprey), have been recently discussed [for review: Ref. (5, 8)].

In jawless vertebrates, three GnRH decapeptides were described and named as lamprey GnRH-I, -II, and -III. Lamprey GnRH-I and -III were initially proposed to be grouped in a type 4 GnRH clade (16). A few years later, Kavanaugh et al. supported this classification and considered lamprey GnRH-II as a paralog of all gnathostome GnRHs (12). With the availability of whole genome sequences and subsequent syntenic analysis, a new view of the vertebrate GnRH relationships emerged that included four paralogy groups with type 4 being lost in the vertebrates, lamprey GnRH-II grouping with the type 2 GnRHs and lamprey-I and -III grouping with type 3 GnRHs (5, 8).

Back to 1984, cloning of human GnRH cDNA revealed that GnRH is synthetized as a preprohormone consisting of a signal peptide, the GnRH peptide itself, a conserved GKR amidation and proteolytic processing site, and 56 amino acids (aa)—GnRH-associated peptide denominated GAP (17). The authors raised the question of the possible biological function of GAP as hypophysiotropic factor or as carrier protein for GnRH.

A breakthrough was made 1 year later by Nikolics et al., who synthesized human GAP and revealed a potent activity as a prolactin (PRL)-inhibiting factor, on cultured rat pituitary cells (18). They also reported that the inhibition of PRL basal secretion obtained by GAP was comparable to that reported for dopamine. Two years later, it was shown that GAP was co-secreted with GnRH into the hypophyseal portal blood of ovariectomized sheep (19). The PRL inhibitory effect was also observed *in vivo* in rats (20), humans (21), and in rabbits during the process of raising GAP antisera (18). In addition, some reports showed that GAP could stimulate gonadotropins release in rats *in vitro* and *in vivo* (18, 22–26). Taken together, these results indicated that GAP could have a physiological significance in the regulation of pituitary function in mammals.

However, there are also reports where these GAP effects were not observed, as for instance in human pituitary prolactinoma *in vitro* (27) or in sheep *in vivo* (28). To the best of our knowledge, a study by Planas et al. (29), on the effects of human GAP on PRL release in tilapia, has been the only attempt so far to investigate the possible biological function of GAP in non-mammalian vertebrates.

In contrast to GnRH, the GAP function has not been deeply studied and there have been almost no research reports since the 1990s. As an example, a PubMed^1^ browse for “GnRH” retrieved 39,186 items, meanwhile the search for “gonadotropin-releasing hormone-associated peptide” retrieved 65 items where some of them were related to the use of antibodies raised against GAP for GnRH localization studies (1st May 2017).

While a number of recent works have addressed the functions and evolution of GnRH (5, 30–34), no attention has been paid to GAP function(s). From a comparative point of view, the observation by various authors of a very low conservation of GAP sequences among mammals, and furthermore among vertebrates, as compared to the high conservation of GnRH sequences, did not encourage deeper investigation. Based on the poorly conserved sequences, it was assumed that GAP had no other functions than the correct folding of the GnRH precursor [for instance Ref. (35)].

In this conceptual frame, and considering that the spatial conformation of a protein may define its function (36, 37), we focused our attention on the three-dimensional (3D) structure of GAP throughout the vertebrate lineage.

## Materials and Methods

### GAP Sequences Search and Comparison

Amino acid sequences of GAP from each prepro-GnRH type were obtained from the protein NCBI database^2^ or from the Ensembl Genome Browser.^3^ Only those complete available sequences (GnRH + GAP sequences) were considered for this study, including representative species of the different vertebrate groups (accession numbers are provided in Table S1 in Supplementary Material): non-jawed fish (lamprey), chondrichtyan (elephant shark), teleosts (eel, anchovy, zebrafish, medaka, salmon, whitefish, and nile tilapia, among others), non-teleost actinopterygians (sturgeon and spotted gar), basal sarcopterygian (coelacanth), amphibian (clawed frog and bullfrog), sauropsids (turtle, gecko, alligator, and birds), mammals (koala, giant panda, sheep, rat, and human). In all cases, GAP sequences were delimited between the dibasic site for proteolytic processing after the GnRH sequence and the stop codon of the open reading frame.

Pairwise amino acid sequence identity (% of identical amino acids at the same position) and similarity (% of amino acids, identical or with similar physicochemical properties at the same position) were calculated using the Sequence Identity And Similarity (SIAS) server^4^ with a previous alignment with the program tool MUSCLE (38) as it is described in the next section.

### GAP Phylogenetic Analysis

Amino acid sequences of 69 chordate GAP (68 sequences from vertebrate species and one sequence from amphioxus, *Brachiostoma floridae*, used as outgroup) were retrieved from NCBI and Ensembl databases. The 69 GAP sequences were aligned using MUSCLE (38) included in SeaView (version 4.6.1) and manually adjusted (Figure S1 in Supplementary Material). The JTT (Jones, Taylor, and Thornton) protein substitution matrix of the resulting alignment was determined using the Protest software (39). Phylogenetic analysis of the GAP sequence alignment was performed using the Maximum Likelihood method with 1,000 bootstrap replicates^5^ [RaxML software (40)].

### Prediction of GAP Three-Dimensional Protein Structure

Secondary protein structures of GAP variants from the different vertebrates were modeled using the I-TASSER server, an automated protein-modeling server from the Zhang Lab at the University of Michigan^6^ (41). Only models with the *C*-score between 2 and −4 were considered. The visualization of the predicted three-dimensional structures was performed using the Jmol software.^7^

## Results

### GAP Sequences Comparison

We compared the lengths and amino acid sequences of the GAP associated to the three GnRH types present in gnathostomes, named in this study as GAP1, GAP2, and GAP3, and of the GAPs associated to the lamprey GnRHs named as GAP-I, -II, and -III. GAP1 sequences analyzed presented a length between 53 and 63 aa, being 56 aa, as in human, the most common length. In the case of GAP2, non-mammalian vertebrate sequences presented a length between 45 and 50 aa, being 49 aa the most common length. However, mammalian GAP2 sequence lengths presented important variations among the analyzed species: koala 39 aa, human 77 aa, giant panda 80 aa, and sheep 84 aa. In case of teleost GAP3, lengths varied between 46 and 58 aa. These observations are displayed in Table S2 in Supplementary Material. Lamprey GAP-I, -II, and -III presented 58, 69, and 56 aa, respectively.

Amino acid SIAS was analyzed, within each GAP type (Table S3 in Supplementary Material). For GAP1, mammalian sequences presented relatively high sequence identity (55–75%) and similarity (72–85%). Lower sequence identity was observed between mammalian and non-mammalian sequences, with the majority of values lower than 50% (gray boxes in Table S3 in Supplementary Material), the lowest percentages being observed with some teleost species (as low as 9% identity between rat and medaka). Low percentages of identity were also observed among teleosts themselves (11–69%). Similarity scores were higher than identity ones, with 32% similarity between rat and medaka, and 29–82% between teleost species studied.

For GAP2, important divergences were observed for some mammalian sequences such as between human and koala (15% identity and 43% similarity). Among teleost species analyzed, GAP2 sequences presented higher percentages of identity (47–98%) and similarity (83–100%) than for GAP1. As compared to GAP2 sequences from various gnathostomes, lamprey GAP-II presented low percentages of identity (3–22%) and similarity (12–50%).

In the case of teleost GAP3, identity and similarity percentages ranged between 18 and 92% and 37 and 97%, respectively, reflecting large sequence variations, as for GAP1. Lamprey GAP-I and -III paralogs shared 66% identity and 77% similarity. As compared to teleost GAP3 sequences, they presented low percentages of identity (7–15%) and similarity (14–36%).

### GAP Phylogenetic Analysis

**Figure 1:**
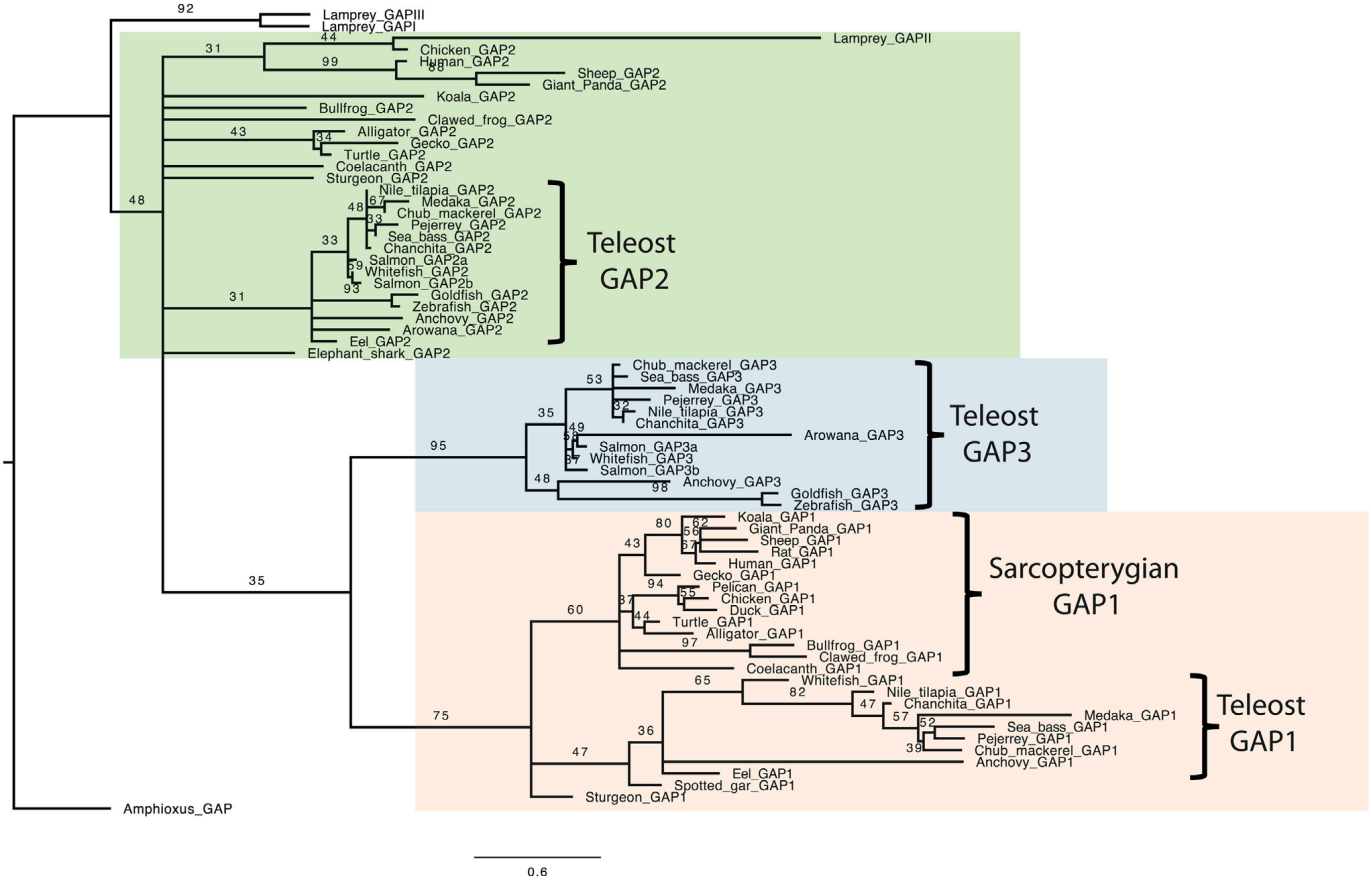
Consensus phylogenetic tree of vertebrate GnRH-associated peptide (GAP). Phylogenetic analysis of 69 GAP amino acid sequences was performed using Maximal Likelihood, with 1,000 bootstrap replicates. The number shown at each branch node indicates in percentage the bootstrap value. Only values above 30% are indicated. The tree is rooted with a non-vertebrate chordate (Amphioxus) GAP sequence used as an outgroup. Alignment and sequence references are given in Figure S1 and Table S1 in Supplementary Material, respectively.

In order to further compare GAP amino acid sequences, a phylogenetic analysis was performed, based on a 68 vertebrate GAP amino acid sequence alignment (Figure S1 in Supplementary Material), with the amphioxus GAP used as outgroup. This alignment was performed using the MUSCLE tool, and a phylogenetic tree was generated using Maximum of Likelihood method with 1,000 bootstrap replicates (Figure 1). As expected, this analysis showed large sequence variations, with only a few nodes being supported by bootstrap values over 50. The analysis clustered all analyzed GAP1 sequences in a well-supported clade (boostrap value of 75). In this group, sarcopterygian sequences and teleost sequences clustered in two distinct clades supported by boostrap values 60 and 36, respectively. Teleost GAP1 clade showed large sequence variations, as illustrated by long branches among species. GAP2 sequences (including gnatosthome GAP2 and lamprey GAP-II) did not cluster in a single clade, indicating major divergences throughout vertebrate radiation. Teleost GAP2 sequences formed a clade with a boostrap value of 31. This GAP2 teleost clade showed short branch lengths reflecting some sequence conservation. Lamprey GAP-II clustered with some of the gnathostome GAP2 sequences (chicken, giant panda, sheep, and human; boostrap value of 31) in a group with long branches, suggesting large sequence variation among these species. Concerning teleost GAP3 sequences, they clustered in a well-supported clade (bootstrap value of 95). Some sequences, like GAP3 of arowana, goldfish, and zebrafish, presented longer branches than the other teleost GAP3, indicating some large sequence variations among this group. Teleost GAP3 appeared as a sister clade to gnathostome GAP1 (bootstrap value 35). In contrast, lamprey GAP-I and -III, while being possibly related to gnathostome GAP3 (5, 8), did not cluster with any specific GAP clade and were positioned together at the base of the phylogenetic tree.

### Prediction of the Three-Dimensional Protein Structure

**Figure 2:**
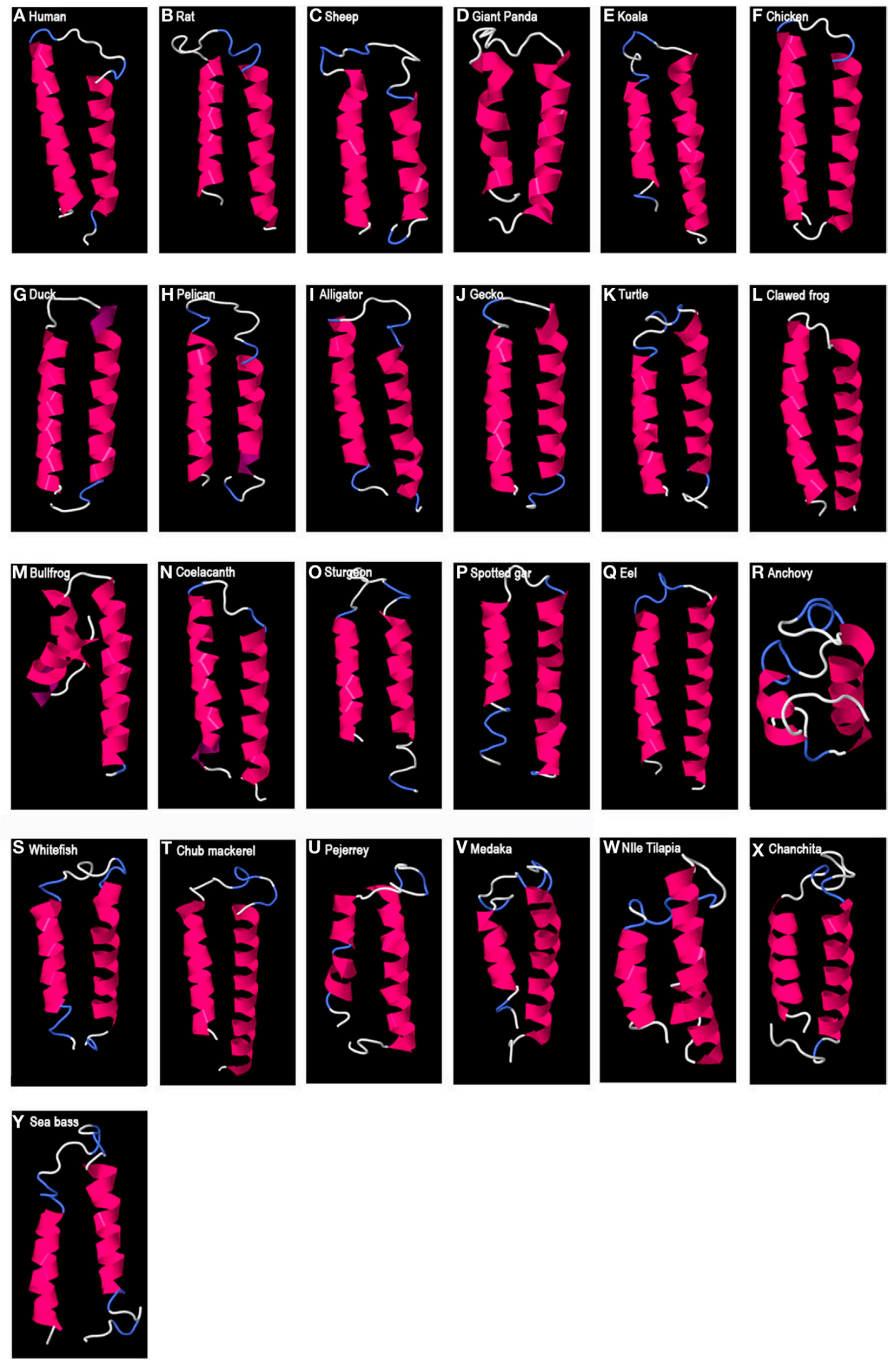
Predicted 3D structure of gnathostome GAP1. Models from different representative vertebrates are shown: mammals **(A–E)**, sauropsids **(F–K)**, amphibians **(L,M)**, coelacanth **(N)**, non-teleost actinopterygians **(O,P)**, and teleosts **(Q–Y)**. Models predicted in I-TASSER server with a *C*-score between 2 and −4 were presented. In all the models, the C-terminal is oriented toward the right. In pink appears α-helix, in violet 3_10_-helix, in white loops, and in blue β-turns.

Predicted secondary protein structures of GAP variants were obtained using the I-TASSER server. For GAP1, a 3D structure characterized by two alpha helices separated by a loop was obtained (Figure 2). This helix-loop-helix (HLH) structure was predicted for all GAP1 sequences analyzed. However, some variations were observed in the length of the alpha helices, as well as in the length of the loop. As compared to the 9 aa loop in human, the number of aa involved in the loop varied from 3 aa, in case of the bullfrog, to 15 aa as in medaka, Nile tilapia, and sheep, and up to 17 aa in seabass and 21 aa in anchovy. The number of aa involved in the alpha helices (24 aa and 17aa in human) varied: in the N-terminal helix from 8 aa in anchovy to 24 aa in human or 23 aa in chicken and in the C-terminal helix from 15 aa in anchovy to 28 aa in the eel.

**Figure 3:**
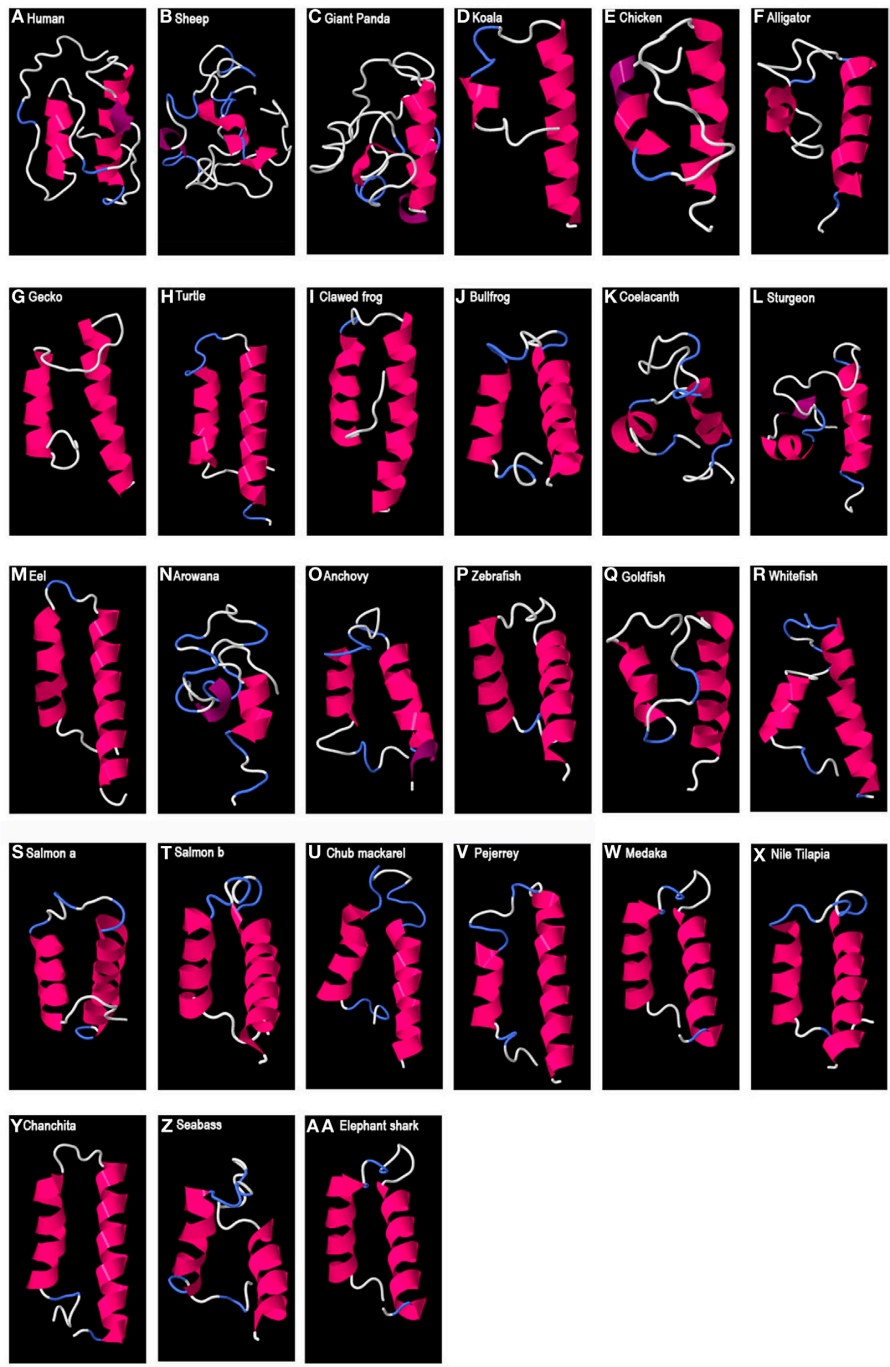
Predicted 3D structure of gnathostome GAP2. Models from different representative vertebrates are shown: mammals **(A–D)**, sauropsids **(E–H)**, amphibians **(I,J)**, coelacanth **(K)**, non-teleost actinopterygians **(L)**, teleosts **(M–Z)**, and chondrichthyes **(AA)**. Models predicted in I-TASSER server with a *C*-score between 2 and −4 were presented. In all the models, the C-terminal is oriented toward the right. In pink appears α-helix, in violet 3_10_-helix, in white loops, and in blue β-turns.

In the case of GAP2-predicted 3D structure (Figure 3), an HLH structure, with long alpha helices, was observed in some species such as in a chondrichtyan (elephant shark), various teleosts, amphibians (clawed frog and bullfrog), and some sauropsids (gecko and turtle). These structures presented a loop of between 4 and 10 aa, depending on the species. However, some other GAP2 studied only showed one, or very short or no alpha helix such as in some mammals (sheep, koala, and giant panda), in a sauropsid (alligator), in a basal sarcopterigyan (coelacanth), in a chondrostean (sturgeon), and in a teleost (arowana). In human, two small alpha helices (9 aa and 14 aa) separated by a large loop (22 aa) were observed.

**Figure 4:**
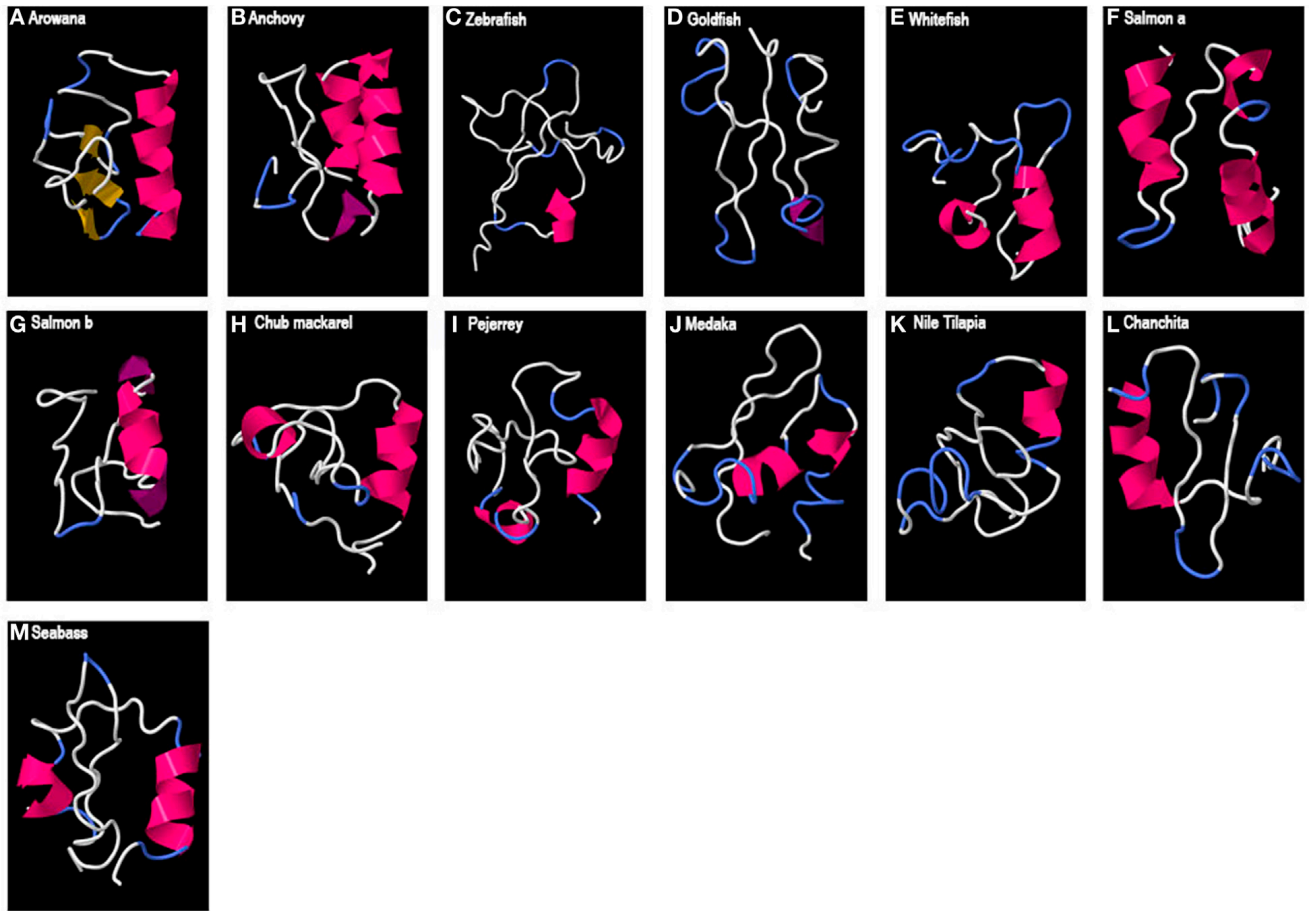
Predicted 3D structure of teleost GAP3. Models from different teleost fish are shown (A–M). Models predicted in I-TASSER server with a *C*-score between 2 and −4 were presented. In all the models, the C-terminal is oriented toward the right. In pink appears α-helix, in violet 3_10_-helix, in yellow β-sheets, in white loops, and in blue β-turns.

Finally, in the case of teleost GAP3 (Figure 4), either no 3D organized structure could be predicted, being only random coils (zebrafish or goldfish) or small alpha helices (most around 9 aa) were observed. For the arowana, β-sheet structures were also obtained.

**Figure 5:**
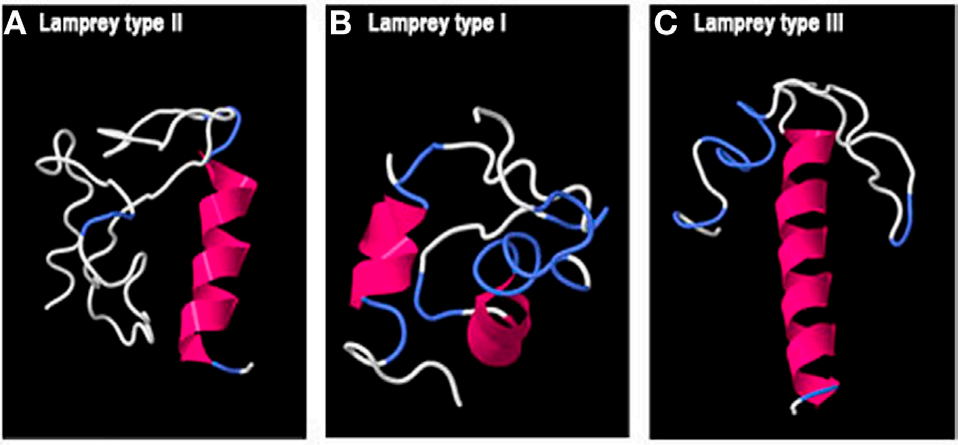
Predicted 3D structure of lamprey GnRH-associated peptides (GAPs). Models from lamprey GAP-II **(A)**, GAP-I **(B)**, and GAP-III **(C)** are shown. Models predicted in I-TASSER server with a *C*-score between 2 and −4 were presented. In all the models, the C-terminal is oriented toward the right. In pink appears α-helix, in white loops, and in blue β-turns.

Concerning lamprey GAPs, two small alpha helices with long loops were predicted for GAP-I, while lamprey GAP-II and -III presented a single alpha helix (Figure 5).

In the sequence alignment (Figure S1 in Supplementary Material), two cysteine residues were present and conserved in all gnathostome GAP2 sequences analyzed, except in mammalian GAP2 where both cysteine residues have been lost. When an HLH structure was observed, the second cysteine was predicted to be located in the loop of the GAP2 HLH, while the first one was mostly observed in the N-terminal helix but in some cases in the loop, depending of the length of the helices described. Gnathostome GAP1 sequences, including human GAP1, had conserved the second cysteine (except for two teleost species, medaka and anchovy). This cysteine was predicted to be located in the loop of the GAP1 HLH structure with the exception of the bullfrog where it was predicted in the end of the N-terminal helix. Teleost GAP1 had also conserved the first cysteine residue, as for GAP2, with the exception of the eel. In contrast, no cysteine was present in any teleost GAP3 sequences analyzed. Lamprey GAP-II, -I, and -III presented the two cysteine residues as in gnathostome GAP2, while lamprey GAP-II presented an additional three cysteine residues.

## Discussion

Elucidation of GAP activity as a potential regulator of the pituitary gland function has not been taken into consideration for the last 20 years. Furthermore, comparison of GnRH precursor sequences between vertebrates led authors to conclude the low conservation of the GAP sequences suggesting that GAP may only participate in the folding and processing of GnRH prohormone, without any specific and/or conserved physiological function [for instance Ref. (35, 42)]. However, our present results revealed that, in spite of low SIAS, GAP1 sequences present a remarkably conserved predicted 3D structure between vertebrates that could define possible biological activities and neuroendocrine functions.

### Conservation of GAP Size Range

We compared GAP sequences from representative species of various vertebrate groups. In the present study, we used the nomenclature GAP1, GAP2, and GAP3, referring to the corresponding three types of GnRH (GnRH1, GnRH2, and GnRH3) in gnathostomes, according to the current GnRH classification (5–9). For lamprey, we used GAP-I, GAP-II, and GAP-III, referring to the corresponding initial lamprey GnRH nomenclature (GnRH-I, -II, and -III). As highlighted in the “Introduction” section, recent evolutionary scenarii are grouping lamprey GnRH-II with gnathostome GnRH2 and lamprey GnRH-I and -III with gnathostome GnRH3, respectively (5, 8, 9).

We compared the lengths of GAP peptides as a first indicator of their variations. GAP1 sequences present a length of 56 aa in the majority of the species studied, as in human, with some deletions or insertions leading to a maximum of 3 aa length variation in some species. A similar situation occurs for most GAP2 with a length around 49 aa. However, it is interesting to note that in mammals, where GnRH2 is non-functional in some species [for review see Ref. (13, 14)], the length of the GAP peptide is very variable probably suggesting more evolutionary freedom to mutations. For GAP3, as evaluated in teleosts, lengths varied from 46 to 58 aa. In lamprey, GAP-I and -III, which are encoded by two GnRH genes resulting from a lamprey-specific duplication, present a similar length (58 and 56 aa), while lamprey GAP-II is longer with 69 aa. Altogether, these results suggest that some evolutionary constraints have limited the range of GAP length variations through vertebrate lineage, in particular for GAP1.

### Poor Conservation of GAP Amino Acid Sequence

In contrast to their similar lengths, GAPs presented large sequence variations, even within each type. In agreement with previous punctual reports [for instance Ref. (35)], comparison of sequences showed low percentages of identity between GAP from different vertebrates and even within a given vertebrate group. This was illustrated by GAP1 sequences, which, despite similar lengths, presented very low percentages of identity between mammals and teleosts, or among teleosts. Some higher percentages were observed in a few cases, such as between GAP2 sequences, when comparing teleosts only. When evaluating similarities (amino acids with similar physico-chemical properties), higher percentages were found than for identity, but they remained low, as shown for instance between mammalian and teleost GAP1.

In order to further analyze GAP sequence divergences, we performed a phylogenetic analysis focused on vertebrate GAP sequences. The phylogenetic tree clustered all GAP1 sequences analyzed, in agreement with the orthology of their corresponding GnRH1 genes. However, in this clade, long branches observed among teleosts reflected important sequence divergences. GAP2 sequences did not form a single clade, indicating major sequence variations among vertebrates. One clade grouped teleost GAP2 sequences, with short branches, suggesting that specific functional constraints may have led to higher conservation of GAP2 sequences in this group. Teleost GAP3 also clustered in one well-supported clade but with short or long branches, depending on the different species represented in this group, suggesting important sequence divergences among teleost species for GAP3. Lamprey GAP-II clustered with some gnathostome GAP2 sequences, in agreement with the proposed orthology (5, 8). In contrast, lamprey GAP-I and -III, proposed as GAP3 orthologs, did not cluster with any specific gnathostome GAP clade; their position at the base of the phylogenetic tree, possibly reflected the conservation of some GAP ancestral features. Overall, this phylogenetic analysis reveals major divergences of GAP sequences throughout vertebrate radiation.

### Striking Conservation of GAP1 Three-Dimensional HLH Structure

In spite of the low sequence identity between vertebrate GAP sequences, we showed that the predicted three-dimensional structures were remarkably conserved in the case of GAP1, with a typical HLH structure. This HLH structure is characterized by two hydrophobic alpha helices linked by a flexible loop.

In 1992, Gupta and Salunke (43) described such an HLH structure for human PRL-inhibiting factor (human GAP1) and related this with the structural motif of DNA-binding proteins that regulate transcription of key developmental genes. Five years later, the same research group demonstrated that the HLH motif is critical for the inhibitory effect of human GAP on PRL secretion, as studied in the rat *in vivo* (26). Shorter synthetic peptides did not show any activity, suggesting the importance of the entire HLH 3D structure for this regulation. The authors concluded that the PRL-inhibiting activity of GAP is defined by its HLH motif, as in the case of the transcription factors regulating developmental genes. Furthermore, homology-based model allowed them to predict the possibility of functional heterodimerization of GAP with ubiquitous transcriptional factors like E-Box binding proteins. To our knowledge, after these pioneer studies and challenging hypothesis, no further investigation was performed on GAP structural properties.

In addition, a conserved single cysteine residue was predicted to be located in the loop of these GAP1 HLH structures, being spatially available to potentially interact by disulfide bridge, possibly in GAP homo- or heterodimerization, or in interactions with other cofactors involved in GAP activity. A cysteine residue located in HLH domain has been proven to play a critical role in the Id2 transcription factor dimerization and biological function (44). Some exceptions were however noticed, this cysteine being missing in medaka and anchovy. In the case of anchovy GAP1, the shorter alpha helices and longer loop may suggest some further divergence, possibly leading to the loss of the typical HLH structure.

In the present study, we revealed that all GAP1 sequences analyzed, from various vertebrates, including various mammals, birds, other sauropsids, amphibians, basal sarcopterygian (coelacanth), teleosts, and other actinopterygians, exhibit an HLH 3D structure. This striking conservation of GAP1 3D structure across vertebrates is consistent with the conservation of GAP1 size range but contrasts with the poor conservation of primary amino acid sequences.

Multiple examples highlight that evolution does favor conservation of structural motifs, protein structures being much more conserved than amino acid sequences [for instance Ref. (45)]. Furthermore, similar 3D functional structural motifs, such as DNA-binding and calcium-binding motifs, may be conserved even in unrelated proteins, supporting the importance of three-dimensional structure over primary amino acid sequence (46). The conservation of GAP1 3D structure thus suggests that evolutionary constraints, related to HLH-defined biological activity, have maintained the HLH 3D structure of GAP1 throughout vertebrate evolution. This fully reopens the question of the biological activity of GAP and of its mechanism of action.

### HLH-Defined Potential Mechanisms of Action of GAP1

As hypothesized by Gupta and Salunke (43), HLH structural motif may confer to GAP a potential function of transcription regulator. Transcription factors with an HLH motif have been proposed to bind as dimers to E-box of different promoters (47). Interestingly, it has been described in rodents that the PRL (48) and the GnRH receptor (GnRHR) promoters (49) present several E-box sequences. For example, the GnRHR promoter presents seven non-canonical E-box sequences where all the regulatory factors are not known yet (50). HLH motif with a basic stretch of residues on the N-terminal may result in the activation of promoters (26), while those regulatory factors that lack the basic region on the N-terminal may carry out negative regulation by interacting with other transcription factors (51–53). As suggested by Chavali et al., this characteristic may explain why human GAP1, which present an HLH motif without a basic region on the N-terminal, acts as an inhibitory PRL factor. In the alignment performed in this study neither of the GAP1 sequences analyzed present a stretch of basic aa on the N-terminal, which may suggest a conservative property. However, despite these pioneering hypotheses, no further investigation directly addressed the potential transcription regulatory mechanism of action of GAP.

Besides this potential transcription regulatory activity, HLH motif may also display calcium-binding-related activity. The calcium-binding sites of the HLH proteins require a polypeptide chain segment with 10–12 residues (54). It is within this loop region that calcium is ligated to oxygen atoms at defined conserved positions (54, 55). Some studies reported an inhibition on PRL release concomitant with a decrease in intracellular Ca^2+^ levels in pituitary cells treated with GAP (56–58). In the present study, we have obtained HLH structures for GAP1 with a loop of similar length to that described for calcium-binding proteins; however, we also found diversity in the aa sequences of the loop. Nevertheless, we cannot rule out a possible binding calcium activity.

As already mentioned, in some mammalian species, it was described that GAP is co-secreted with GnRH into the hypophyseal portal blood (19, 59), reaching the adenohypophyseal cells. However, a GAP receptor has not been described yet, which also contributed to the lack of interest of the scientific community. Nevertheless, it should be taken into account that this is not an impediment for a possible function of GAP, since internalization of this protein could be considered. Chen et al. (60) proposed that HLH proteins possess a cell penetration property and the internalization mechanism would be primary by a type of macropinocytosis. In addition, it has been already reported that a pharmacological molecule with an HLH structure may fuse to the cell membrane or perform a membrane pore entering in this way into the cell (61). Once in the cell, GAP activity could be directly supported by its HLH motif, via binding of calcium, a major intracellular messenger (62), or via heterodimerization with other HLH-regulatory factors involved in gene transcription (26). To our knowledge, no investigation addressed the possible mechanisms of GAP entry into the pituitary cells.

### Potential Hypophysiotropic Functions of GAP1

GAP1 is associated to GnRH1, the major hypophysiotropic GnRH type, responsible for the stimulatory control of reproduction in all gnathostomes, at the exception of some teleost species, such as cypriniformes and salmonids, which have lost this gene type (10). The remarkable conservation of GAP1 3D structure suggests that HLH-defined biological activity of GAP1 may confer neuroendocrine hypohysiotropic functions to GAP1, in coordination with GnRH1, throughout the vertebrate lineage.

In mammals, GAP1 was shown to be released from GnRH1 neuron axonal endings at the level of the median eminence into the portal hypophyseal system, which conveys neuroendocrine hypophysiotropic factors to the pituitary. In mammals, GAP1 hypophysiotropic actions include a potent PRL-inhibiting role and a weaker gonadotropin-stimulating role. In teleosts, differently from mammals and tetrapods, hypohysiotropic neurons directly innervate the adenohypophysis (63); furthermore, the different types of pituitary cells are regionally distributed in the teleost pituitary and receive specific neuronal innervations. As shown by immunocytochemistry, GnRH1/GAP1 neurons innervate the proximal *pars distalis* of the pituitary where LH, FSH cells, and also growth hormone cells are located [for instance Ref. (64, 65)]. As mentioned before, investigation on the effects of GAP in non-mammalian species are critically lacking. Taking into account the GnRH1/GAP1 innervation, future investigation should focus on the potential effects of GAP on gonadotrope and somatotrope cells in teleosts.

### Variable Conservation of HLH 3D Structure in the Other GAPs

In contrast to GAP1, we found no 3D HLH structure for teleost GAP3 sequences analyzed in this study. GAP3 is associated with GnRH type 3, originally discovered in salmon and present in most teleost species studied (66). As GnRH1 neurons, GnRH3 neurons are involved in the hypophysiotropic control of reproduction. Some teleost species are lacking GnRH3 gene, such as elepomorphs (eel), while some others are lacking GnRH1 gene, such as cypriniforms (zebrafish) and salmonids (salmon). A functional equivalence between GnRH1 and GnRH3 neurons has been proposed in these species. However, the present study showing the lack of 3D HLH structure for GAP3 suggests that this equivalence does not apply for GAP possible hypophysiotropic function.

While a conserved 3D structure was predicted for all GAP1 sequences analyzed, and for none of GAP3, GAP2 did not present such a clear scenario. We predicted a typical 3D HLH structure for GAP2 in some of the species studies, including a chondrichtyan (elephant shark), some, but not all, teleosts, amphibians (clawed frog and bullfrog), some sauropsids (gecko and turtle), while in other species, only one or no helix was predicted. In the case of human, two alpha helices were predicted but with distinct features such as large number of amino acids in the loop and in the amino- and carboxy-termini tails. GAP2 is associated with GnRH2, the “midbrain” GnRH type discovered in birds and retrieved in all vertebrates. Differently from the canonical hypophysiotropic function of GnRH1, the roles of GnRH2 are less known and may include various and species-specific brain functions in metabolism, appetite, and sexual behavior, among others (10, 67). The large variation in GAP2 sequences and 3D structures across vertebrates may be related to the plasticity of GnRH2 neuron functions according to vertebrate species. However, the fact that an HLH structure was found in some species, as in the majority of teleosts, is an interesting feature that deserves further consideration.

Concerning lamprey GAPs, no 3D HLH structure was observed. According to the recent evolutionary scenario of vertebrate GnRH genes (5, 8), we can assume that lamprey GAP-II would be related to gnathostome GAP2, for which an HLH structure could be predicted in some but not all species, and lamprey GAP-I and -III to gnathostome GAP3, for which no HLH structure could be observed.

## Conclusion

As supported by the poor conservation of its primary amino acid sequence, GAP function has been considered to be restricted to the folding, processing, and carrying of GnRH, contributing to the disinterest toward this neuropeptide for the past 20 years. The present study revealed a striking conservation of GAP1 3D HLH structure throughout the vertebrate lineage, which allows us to reopen the question of the possible biological activity and neuroendocrine functions of the GAP peptide itself. As a matter of fact, if a conserved 3D structure is necessary for the folding and processing of GnRH, how this function would be achieved in the cases of lamprey GnRHs, GnRH3, and some GnRH2? Conversely, if only the presence of an undefined amino acid sequence downstream the GnRH peptide in the preprohormone would be necessary, then a question arises: why there has been a selective pressure to conserve a 3D HLH structure for more than 400 million years in GAP1? The present findings provide evolutionary and structural bases to promote new research avenues on GAP molecular mechanisms of action defined by its HLH structure, such as cell entry, heterodimerization, gene transcription regulation, and calcium binding, as well as on the final and species-specific biological effects of GAP, across vertebrates.

## Author Contributions

DPS and A-GL performed the analyses and did the figures. All authors contributed to the design of the work, interpretation of the results, and writing of the manuscript, and all approved the final version of the manuscript.

## Conflict of Interest Statement

The authors declare that the research was conducted in the absence of any commercial or financial relationships that could be construed as a potential conflict of interest.

## Footnotes

^1^http://www.ncbi.nlm.nih.gov/pubmed.

^2^http://www.ncbi.nlm.nih.gov/protein.

^3^http://www.ensembl.org/index.html.

^4^http://imed.med.ucm.es/Tools/sias.html.

^5^www.phylo.org.

^6^http://zhanglab.ccmb.med.umich.edu/I-TASSER/.

^7^http://jmol.sourceforge.net/index.en.html.

## Supplementary Material

Figure S1 Alignment of vertebrate GAP sequences. The alignment was performed using muscle included in SeaView (version 4.6.1) and manually adjusted. The first * indicates the position of the cysteine residue conserved in GAP2 sequences, except in mammals, in most of the teleost GAP1 sequences and in lamprey GAP-I, -II, and -III. The second * indicates the position of the cysteine residue conserved in GAP2 sequences, except in mammals, in all GAP1 sequences, except medaka and anchovy, and in lamprey GAP-I, -II, and -III. This alignment was used for the phylogenetic analysis (Figure 1) and for the calculation of identity and similarity percentages (Table S3 in Supplementary Material).

Table S1 Vertebrate species and accession numbers of the complete prepro-GnRH sequences used to analyze GAP sequences. Lines divide the different vertebrate groups. GB. GenBank database; EN, Ensembl Genome Browser. *Sequence obtained from Ref. (68), which is not available in the data bases consulted.

Table S2 Tables showing the amino acid (aa) lengths of GAP1, GAP2, and GAP3 from various gnathostome species. Bold indicates the more representative length in all species analyzed.

Table S3 Amino acid sequence identity (*I*%) and similarity (*S*%, in bold) within each GAP type. Gray indicates those values, both identity and similarity, lower than 50%.
