# Supplementary material for: Conservation of Three-Dimensional Helix-Loop-Helix Structure through the Vertebrate Lineage Reopens the Cold Case of Gonadotropin-Releasing Hormone-Associated Peptide
Source: Front Endocrinol (Lausanne). 2017 Aug 22;8:207. doi: 10.3389/fendo.2017.00207 (PMC5572233; doi:10.3389/fendo.2017.00207)
Supplement: Figure S1 — Alignment of vertebrate GAP sequences. The alignment was performed using muscle included in SeaView (version 4.6.1) and manually adjusted. The first * indicates the position of the cysteine residue conserved in GAP2 sequences, except in mammals, in most of the teleost GAP1 sequences and in lamprey GAP-I, -II, and -III. The second * indicates the position of the cysteine residue conserved in GAP2 sequences, except in mammals, in all GAP1 sequences, except medaka and anchovy, and in lamprey GAP-I, -II, and -III. This alignment was used for the phylogenetic analysis (Figure 1) and for the calculation of identity and similarity percentages (Table S3 in Supplementary Material). [file Image_1.PDF]

```
                    1                                                        *
Human_GAP1          ---------- ---------- ---------D AE-NLIDSFQ --EIVKEV-G QLA--ETQRF
Rat_GAP1            ---------- ---------- ---------N TE-HLVDSFQ --EMGKEE-D QMA--EPQNF
Sheep_GAP1          ---------- ---------- ---------N AE-NVIDSFQ --EIAKEV-D QPV--EPKHC
Koala_GAP1          ---------- ---------- ---------D AD-NLIDSFQ --EMADEG-N QLA--EPQRF
Giant_Panda_GAP1    ---------- ---------- ---------N AE-KLIDSFQ --EIAKEL-D QGA--EPQHL
Chicken_GAP1        ---------- ---------- ---------N AE-NLVESFQ --EIANEM-E SLG--EGQKA
Duck_GAP1           ---------- ---------- ---------N VD-NLGELFQ --EIANDM-E KIG--EVQKT
Pelican_GAP1        ---------- ---------- ---------N AE-NLVESFQ --ETANEM-E KSG--EVQKT
Alligator_GAP1      ---------- ---------- ---------N AE-NVVESFQ --QMASDM-E KFG--EMQQF
Gecko_GAP1          ---------- ---------- ---------D AE-NLIESFQ --EIANEV-D KVG--ELQHL
Turtle_GAP1         ---------- ---------- ---------D AE-NLVESFQ --EIASEM-E KIG--EMQHF
Clawed_frog_GAP1    ---------- ---------- ---------D AE-SLQDMYP --ETPNEV-P LFP--ELERL
Bullfrog_GAP1       ---------- ---------- ---------E VE-SLQESYA --EVPNEV-- SFT--ELQHL
Coelacanth_GAP1     ---------- ---------- ---------N SE-HLMESFQ --DITNNA-E RPV--EPEQS
Sturgeon_GAP1       ---------- ---------- ---------E TE-TLLDTLQ --EIA-DI-E KLD--TGDHS
Spotted_gar_GAP1    ---------- ---------- ---------E VE-SLQDTLQ --DIAEEV-R KLD--AIRQP
Eel_GAP1            ---------- ---------- ---------G AD-SLQDTLQ --DIIEEL-Q KLD--TSSLP
Medaka_GAP1         ---------- ---------- ---------E LK-YFPNTLE --NQIRLL-N SNT--PCSDL
Sea_bass_GAP1       ---------- ---------- ---------E LD-GLSETLG -NQIVGSF-P HVA-TPCRVL
Chanchita_GAP1      ---------- ---------- ---------D LD-TFSDALG --NMVEEF-P RVE-APCSVF
Whitefish_GAP1      ---------- ---------- ---------A TG-SLSDTQD --NMAEDL-L KID-PSCSLF
Anchovy_GAP1        ---------- ---------- ---------E AD-SPSES-Q ---VMEGLPR RGG-ARCGSD
Chub_mackerel_GAP1  ---------- ---------- ---------E LD-SLSDTMD --DVVEGF-P QVD-TPCSFL
Nile_tilapia_GAP1   ---------- ---------- ---------D LD-NFSDTLG --NMVEEF-P RVE-APCSVF
Pejerrey_GAP1       ---------- ---------- ---------D LD-TFSDTLD --NVVEGF-P HMD-APCRVV
Goldfish_GAP3       ---------- ---------- ------SVGE VEATF----- --KMMDAG-D AVLSIP-A--
Chanchita_GAP3      ---------- ---------- ------SVGE LEATI----- --RMMGTG-G VVS-LP-E--
Zebrafish_GAP3      ---------- ---------- ------SVGE MEATF----- --RMLDPG-D TVLSIP-A--
Medaka_GAP3         ---------- ---------- ------SVGE LEATI----- --RMMGTG-R VVS-LP-E--
Sea_bass_GAP3       ---------- ---------- ------SVGE LEATI----- --RMMGTG-E VVS-LP-E--
Whitefish_GAP3      ---------- ---------- ------SVGE LEATI----- --RMMDTG-G EVA-LP-E--
Salmon_GAP3a        ---------- ---------- ------SVGE LEASI----- --KMMDTG-G VVA-LP-E--
Salmon_GAP3b        ---------- ---------- ------SVGE LEATI----- --RMMDTG-G VMV-LP-E--
Anchovy_GAP3        ---------- ---------- -----SIGGE LEATF----- --RMMDAG-D TL--IP-L--
Arowana_GAP3        ---------- ---------- ------STGD TEAKV----- --KMMDSG-D LVT-FF-E--
Chub_mackerel_GAP3  ---------- ---------- ------SVGE LEATI----- --RMMGTG-G VVS-LP-E--
Nile_tilapia_GAP3   ---------- ---------- ------SVGE LEATI----- --RMMGTG-E VVS-LP-D--
Pejerrey_GAP3       ---------- ---------- ------SVGE LEATI----- --RMMGTG-G VVS-LP-E--
Medaka_GAP2         ---------- ---------- ---------E LD-SF----- -----EVS-E EMK-L-CETG
Goldfish_GAP2       ---------- ---------- ---------E ID--VY---- --DPSEVS-E EIK-L-CNAG
Zebrafish_GAP2      ---------- ---------- ---------E ID--LY---- --DTSEVS-E EVK-L-CEAG
Chanchita_GAP2      ---------- ---------- ---------E LD-SF----- --GTSEIS-E EIK-L-CEAG
Sea_bass_GAP2       ---------- ---------- ---------E LD-SF----- --GTSEIS-E EIK-L-CEAG
Whitefish_GAP2      ---------- ---------- ---------E LD-SF----- --TTSEIS-E EIK-L-CEAG
Salmon_GAP2a        ---------- ---------- ---------E LD-SF----- --TTSEIS-E EIK-L-CEAG
Salmon_GAP2b        ---------- ---------- ---------E LD-SF----- --TTSEIS-E EIK-L-CEAG
Anchovy_GAP2        ---------- ---------- ---------D VD-TF----- -NSAQ-VS-E EIK-L-CEAG
Arowana_GAP2        ---------- ---------- ---------E LN-SL----- --TASEVS-G KIK-L-CEDR
Chub_mackerel_GAP2  ---------- ---------- ---------E LD-SF----- --GTPEIS-E EIK-L-CEAG
Nile_tilapia_GAP2   ---------- ---------- ---------E LD-SF----- --GTSEIS-E EIK-L-CEAG
Pejerrey_GAP2       ---------- ---------- ---------E LD-SF----- --STSEIS-E ENK-L-CEAG
Eel_GAP2            ---------- ---------- ---------E LD-SL----- --TTAEVL-D EIK-L-CDGG
Sturgeon_GAP2       ---------- ---------- ---------E LE-GL----- --QSPEDS-D EVK-L-CDGD
Coelacanth_GAP2     ---------- ---------- ---------E LA-IP----- --QTPEVS-E EIK-L-CDGE
Clawed_frog_GAP2    ---------- ---------- ---------Q LD-TR----- --SFPEIS-D ELK--PCEGE
Bullfrog_GAP2       ---------- ---------- ---------E LD-MP----- --ASPEVS-E EIK-L-CEGE
Gecko_GAP2          ---------- ---------- ---------E VD-LS----- --QSPEVS-E DIK-L-CDGD
Turtle_GAP2         ---------- ---------- ---------E LD-LS----- --QAPEAS-E EIK-L-CDGE
Alligator_GAP2      ---------- ---------- ---------E LD-LS----- --QAPQAS-E EIK-I-CGGE
Chicken_GAP2        ---------- ---------- ---------D LS-AP----- --QVP-A--- ALR--PCPTP
Koala_GAP2          ---------- ---------- ---------A LD-EI----- --PGLEAS-E EGK-L-WDGG
Human_GAP2          ---------- ---------- ---------A LS-SA----- --QDPQ---N ALR-PPAGSP
Giant_Panda_GAP2    ---------- ---------- ---------A SS-SA----- --QHPQ---H APR-L-LGRV
Sheep_GAP2          ---------- ---------- ---------A SS-LP----- --RDPQ---H PPR-PP----
Elephant_shark_GAP2 ---------- ---------- ---------E LG-QA----- --QTPEVS-E VFQ-L-CEGD
Lamprey_GAPI        ---------- ---------- ---------D LEVSHTRELE --QELEPPSN AFE---CDGP
Lamprey_GAPII       GVQEPPRASY ENVSPSDGSP FTPVSSGLQV ADW-H----- --VVCSRSPN GFS--GC-AM
Lamprey_GAPIII      ---------- ---------- ---------D LEA-MRPLLE --EELEAPNS AFE---CDGP
Amphioxus_GAP       ---------- ---------- ----RADSSE LL-TPHAAAD SVSAAEVY-D ASE-----GS
```

```
                     61*
Human_GAP1           EC-TT-HQPR -SPL-R-DL- KGAL---ESL IE-------- ---------- -EETGQKKI-
Rat_GAP1             EC-TV-HWPR -SPL-R-DL- RGAL---ERL IE-------- ---------- -EEAGQKKM-
Sheep_GAP1           GC-IV-HQSH -SPL-R-DL- KEAL---ESL IE-------- ---------- -EETGQRKI-
Koala_GAP1           EC-TI-HQPR -SPL-R-DL- KGVL---ASL IE-------- ---------- -GEAGRKKA-
Giant_Panda_GAP1     EC-TI-HQPQ -TPL-R-DL- KGAL---ESL IE-------- ---------- -EENGQKRI-
Chicken_GAP1         EC-PG-SYQH -PRL-S-DL- KETM---ASL IE-------- ---------- -GEARRKEI-
Duck_GAP1            EC-PG-SYQH -PQF-T-DL- KEAM---ASL IE-------- ---------- -GEARRKKI-
Pelican_GAP1         EC-PG-LRQH -SRF-S-DL- KEAM---ESL IE-------- ---------- -GEARRKKI-
Alligator_GAP1       ECSSG-PHQP -SKL-S-DL- KKAL---ASL IE-------- ---------- -GEAGRKKT-
Gecko_GAP1           EC-TA-SQQR -PTL-Q-GL- KGAL---ASL ID-------- ---------- -RETGQKKI-
Turtle_GAP1          EC-TG-PHQR -SML-S-GL- KGAL---ASL IE-------- ---------- -GDAGRKKI-
Clawed_frog_GAP1     EC----SVPQ -SRL-N-VL- RGAL---MSW LD-------- ---------- -GEN-RKKI-
Bullfrog_GAP1        EC----SIPQ -NRI-S-LV- RDAL---MNW LE-------- ---------- -GENARKKI-
Coelacanth_GAP1      EC----SHPQ QSRI-N-LL- KGAL---ANL IE-------- ---------- -GEAGKKKM-
Sturgeon_GAP1        EC-AL-SSQR -SQL-S-DL- KGVL---ARL VG-------- ---------- -GESARKKI-
Spotted_gar_GAP1     GC-AD-VSPQ -SRLSS--L- RELL---ASL AE-------- ---------- -EERGRKNI-
Eel_GAP1             SC-ND-LSPH -ITL-S-SL- KEIL---ANL AD-------- ---------- -RETGRKNI-
Medaka_GAP1          SH-LE-ESSL -AKIYR--I- KGLL---GSV TE-------- ---------- -AKNGYRTYK
Sea_bass_GAP1        GC-AE-ESPF -PKIYR--M- KGFL---DAV TD-------- ---------- -RENGNRTYK
Chanchita_GAP1       GC-AE-ESPF -AKMYR--V- KGLV---GSV TE-------- ---------- -RENGHRTYK
Whitefish_GAP1       GC-AD-VSPH -AKMYR--L- RALL---ASL AD-------- ---------- -RQSGLNNI-
Anchovy_GAP1         T--RD-AANQ -ERPST--L- EQLI----SL MS-------- ---------- -RANEVYD--
Chub_mackerel_GAP1   GC-AE-ESPF -AKIYR--M- KGLF---GSV TN-------- ---------- -RENGHRNYK
Nile_tilapia_GAP1    GC-AE-ESPF -AKMYR--V- KGLL---ASL AE-------- ---------G KTDTGHSRNE
Pejerrey_GAP1        GC-AD-ESPF -AKIYR--M- KGFL---GGV TD-------- ---------- -RENGRRVYK
Goldfish_GAP3        DSPMEQLL-P -IHIV--NEV -DAD---GLP LK-------- ---------- -EQRFPKRRG
Chanchita_GAP3       EASA-QTQER -LRPY--NII -NDD---SSH FD-------- ---------- -GK-------
Zebrafish_GAP3       DSPMEQLS-P -IHIV--NEV -DAE---GLP LK-------- ---------- -GQRFPDRRG
Medaka_GAP3          DASA-QTQER -LRQY--NLI -N-D---GST YF-------- ---------- ---DRKKRFM
Sea_bass_GAP3        EASA-QTQER -LRPY--NVI -NDD---SSH FD-------- ---------- ----RKKRSP
Whitefish_GAP3       ETSA-HVSER -LRPY--DVI -S-------- ---------- ---------- -----KKWMP
Salmon_GAP3a         ETSA-HVSER -LRPY--DVI -L-------- ---------- ---------- -----KKWMP
Salmon_GAP3b         ETGA-HVPER -LRPY--DVM -S-------- ---------- ---------- -----KKRMP
Anchovy_GAP3         -T-A----EK -LQPN--DAI -IDDI--EEN AV-------- ---------- ----RRGRRP
Arowana_GAP3         EAS------P -FVP---ESL -GTNQ--FGS ED-------- --------GG EFTRKRKWM-
Chub_mackerel_GAP3   EASA-QTQEG -LRPY--NVI -NDD---SSH FD-------- ---------- ----RKKRFP
Nile_tilapia_GAP3    EANA-QTQER -LRPY--NII -NDD---SSH FD-------- ---------- ----RKKKVP
Pejerrey_GAP3        EASA-QIQER -FRPY--SVI -NDD---SSH LD-------- ---------- --TWRKKKVL
Medaka_GAP2          ECSY------ -MRPQRRSFL RNIV---LDA LA-------- ---------- -RELQKRK--
Goldfish_GAP2        KCSF------ -LIPQGRNIL KTIL---LDA LT-------- ---------- -RDFQKRK--
Zebrafish_GAP2       KCSY------ -LRPQGRNIL KTIL---LDA LI-------- ---------- -RDFQKRK--
Chanchita_GAP2       ECSY------ -LRPQRRGIL RNIL---LDA LA-------- ---------- -RELQKRK--
Sea_bass_GAP2        ECSY------ -LRPQRRSVL RNII---LDA LA-------- ---------- -RELQKRK--
Whitefish_GAP2       ECSY------ -LRPQRRNIL KNIL---LDA LA-------- ---------- -REFEKRK--
Salmon_GAP2a         ECSY------ -LRPQRRNIL KNII---LDV LA-------- ---------- -REFQKRK--
Salmon_GAP2b         ECSY------ -LRPQRRNIL RNIL---LDA LA-------- ---------- -REFEKIK--
Anchovy_GAP2         ECSY------ -LRPQRRNLL KSIL---LEA LT-------- ---------- -REFQRRK--
Arowana_GAP2         KCSY------ -LRPQQKNIL -TIL---VDA ST-------- ---------- -REFRGKRK-
Chub_mackerel_GAP2   ECSY------ -LRPQRRSFL RNII---LDA LA-------- ---------- -RELQKRK--
Nile_tilapia_GAP2    ECSY------ -LRPQRRSIL RNIL---LDA LA-------- ---------- -RELQKRK--
Pejerrey_GAP2        ECSY------ -LRPQRQNVL RNIV---LDA LA-------- ---------- -RELQKRK--
Eel_GAP2             ECSY------ -LRPQRKSLL KNIL---LDA LA-------- ---------- -REFQRKRK-
Sturgeon_GAP2        ECSY------ -LRHPRKNIL RSIL---ADM LT-------- ---------- -RQMQRKK--
Coelacanth_GAP2      ECTY------ -LRSPRKSIL KEIL---ADI IA-------- ---------- -WQIQKKK--
Clawed_frog_GAP2     SCDY------ -P-MNEMSIL KGLL---TRF LFP------- ---------- -RERQRK---
Bullfrog_GAP2        ECAY------ -LRNPRKNLL KNIL---ADV LA-------- ---------- -RQLQKK---
Gecko_GAP2           DCTY------ -LKIPREKIV TSLL---ADL LA-------- ---------- -KHLQKKK--
Turtle_GAP2          ACAY------ -LRSPRKTIV NTLL---ADL LA-------- ---------- -RQLQKKK--
Alligator_GAP2       ECAY------ -VRSPRMNVV KTLL---ADM LA-------- ---------- -RQLQKKK--
Chicken_GAP2         PC-------- RPLPPMPSTL RAALWRPLEA AL-------- ---------- -RQ-PH----
Koala_GAP2           E--------- ------RSLL KTLL---ADV LA-------- ---------- -QQQQK----
Human_GAP2           VQTA------ ---------- HGL---PSDA LAPLDDSMPW EGRTTAQWSL HRKR-HLART
Giant_Panda_GAP2     LGTA------ -ASSPD-QAA HNL---PSNA LAPPENSVPW EARTTGWWPL -RQKQHLVKT
Sheep_GAP2           ---------- -AQSPG-QIA HTL---PSDA LAWPEDSVPW KSRTMTRWFL -RGKQHLVQT
Elephant_shark_GAP2  DC-AF----- -VRSPRTNLF RSIL---ADL VA-------- ---------- --GRFQKKK-
Lamprey_GAPI         EC-AFSRVPN -TKLIR---- -ELA----SY LS-------- ---------- --QRNYDRKG
Lamprey_GAPII        CCS------- -PGCP--TFL SQV----LEA SL-------- ---------- --GT------
Lamprey_GAPIII       EC-AFARVPT -GELVR---- -EIV----SY LS-------- ---------- --QKNYQRK-
Amphioxus_GAP        E---VTK-ED -FKMAVRTLF R-IL---GDY LQ-------- ---------- --KRTNQN--
```

```
                   121
Human_GAP1           ---------- ---------- -------
Rat_GAP1             ---------- ---------- -------
Sheep_GAP1           ---------- ---------- -------
Koala_GAP1           ---------- ---------- -------
Giant_Panda_GAP1     ---------- ---------- -------
Chicken_GAP1         ---------- ---------- -------
Duck_GAP1            ---------- ---------- -------
Pelican_GAP1         ---------- ---------- -------
Alligator_GAP1       ---------- ---------- -------
Gecko_GAP1           ---------- ---------- -------
Turtle_GAP1          ---------- ---------- -------
Clawed_frog_GAP1     ---------- ---------- -------
Bullfrog_GAP1        ---------- ---------- -------
Coelacanth_GAP1      ---------- ---------- -------
Sturgeon_GAP1        ---------- ---------- -------
Spotted_gar_GAP1     ---------- ---------- -------
Eel_GAP1             ---------- ---------- -------
Medaka_GAP1          ---------- ---------- -------
Sea_bass_GAP1        K--------- ---------- -------
Chanchita_GAP1       K--------- ---------- -------
Whitefish_GAP1       ---------- ---------- -------
Anchovy_GAP1         ---------- ---------- -------
Chub_mackerel_GAP1   K--------- ---------- -------
Nile_tilapia_GAP1    RFL------- ---------- -------
Pejerrey_GAP1        K--------- ---------- -------
Goldfish_GAP3        RV-------- ---------- -------
Chanchita_GAP3       ---------- ---------- -------
Zebrafish_GAP3       RV-------- ---------- -------
Medaka_GAP3          SQ-------- ---------- -------
Sea_bass_GAP3        NK-------- ---------- -------
Whitefish_GAP3       HK-------- ---------- -------
Salmon_GAP3a         HK-------- ---------- -------
Salmon_GAP3b         HK-------- ---------- -------
Anchovy_GAP3         LRRELLD--- ---------- -------
Arowana_GAP3         HQKRII---- ---------- -------
Chub_mackerel_GAP3   HK-------- ---------- -------
Nile_tilapia_GAP3    ---------- ---------- -------
Pejerrey_GAP3        E--------- ---------- -------
Medaka_GAP2          ---------- ---------- -------
Goldfish_GAP2        ---------- ---------- -------
Zebrafish_GAP2       ---------- ---------- -------
Chanchita_GAP2       ---------- ---------- -------
Sea_bass_GAP2        ---------- ---------- -------
Whitefish_GAP2       ---------- ---------- -------
Salmon_GAP2a         ---------- ---------- -------
Salmon_GAP2b         ---------- ---------- -------
Anchovy_GAP2         ---------- ---------- -------
Arowana_GAP2         ---------- ---------- -------
Chub_mackerel_GAP2   ---------- ---------- -------
Nile_tilapia_GAP2    ---------- ---------- -------
Pejerrey_GAP2        ---------- ---------- -------
Eel_GAP2             ---------- ---------- -------
Sturgeon_GAP2        ---------- ---------- -------
Coelacanth_GAP2      ---------- ---------- -------
Clawed_frog_GAP2     ---------- ---------- -------
Bullfrog_GAP2        ---------- ---------- -------
Gecko_GAP2           ---------- ---------- -------
Turtle_GAP2          ---------- ---------- -------
Alligator_GAP2       ---------- ---------- -------
Chicken_GAP2         ---------- ---------- -------
Koala_GAP2           ---------- ---------- -------
Human_GAP2           LL-TAAREPR PAPPSSNKV- -------
Giant_Panda_GAP2     LL-TGRRASR PVALQ----- -------
Sheep_GAP2           LLVSKVEGPH PWPLQGQLRT EVVGG--
Elephant_shark_GAP2  ---------- ---------- -------
Lamprey_GAPI         ALK------- ---------- -------
Lamprey_GAPII        ---------- ---------- -------
Lamprey_GAPIII       VLK------- ---------- -------
Amphioxus_GAP        ---------- ---------- -------
```
